# Functional Characterization of An *Allene Oxide Synthase* Involved in Biosynthesis of Jasmonic Acid and Its Influence on Metabolite Profiles and Ethylene Formation in Tea (*Camellia sinensis*) Flowers

**DOI:** 10.3390/ijms19082440

**Published:** 2018-08-18

**Authors:** Qiyuan Peng, Ying Zhou, Yinyin Liao, Lanting Zeng, Xinlan Xu, Yongxia Jia, Fang Dong, Jianlong Li, Jinchi Tang, Ziyin Yang

**Affiliations:** 1 Guangdong Provincial Key Laboratory of Applied Botany & Key Laboratory of South China Agricultural Plant Molecular Analysis and Genetic Improvement, South China Botanical Garden, Chinese Academy of Sciences, Xingke Road 723, Tianhe District, Guangzhou 510650, China; pqyuan@scbg.ac.cn (Q.P.); yzhou@scbg.ac.cn (Y.Z.); honey_yyliao@scbg.ac.cn (Y.L.); zenglanting@scbg.ac.cn (L.Z.); xxl@scib.ac.cn (X.X.); jyx@scbg.ac.cn (Y.J.); 2 University of Chinese Academy of Sciences, No. 19A Yuquan Road, Beijing 100049, China; 3 Guangdong Food and Drug Vocational College, Longdongbei Road 321, Tianhe District, Guangzhou 510520, China; dongfangxyz@163.com; 4 Tea Research Institute, Guangdong Academy of Agricultural Sciences & Guangdong Provincial Key Laboratory of Tea Plant Resources Innovation and Utilization, Dafeng Road 6, Tianhe District, Guangzhou 510640, China; skylong.41@163.com (J.L.); tangjinchi@126.com (J.T.)

**Keywords:** aroma, *Camellia sinensis*, insect, jasmonic acid, metabolite, tea, volatile

## Abstract

Jasmonic acid (JA) is reportedly involved in the interaction between insects and the vegetative parts of horticultural crops; less attention has, however, been paid to its involvement in the interaction between insects and the floral parts of horticultural crops. Previously, we investigated the *allene oxide synthase 2* (*AOS2*) gene that was found to be the only JA synthesis gene upregulated in tea (*Camellia sinensis*) flowers exposed to insect (*Thrips hawaiiensis* (Morgan)) attacks. In our present study, transient expression analysis in *Nicotiana benthamiana* plants confirmed that CsAOS2 functioned in JA synthesis and was located in the chloroplast membrane. In contrast to tea leaves, the metabolite profiles of tea flowers were not significantly affected by 10 h JA (2.5 mM) treatment as determined using ultra-performance liquid chromatography/quadrupole time-of-flight mass spectrometry, and gas chromatography-mass spectrometry. Moreover, JA treatment did not significantly influence ethylene formation in tea flowers. These results suggest that JA in tea flowers may have different functions from JA in tea leaves and other flowers.

## 1. Introduction

During their evolution, plants are generally exposed to many environmental stresses. Interactions between plants and insects have been intensively studied for many years. In response to insect attack, plants synthesize specialized metabolites or alter their metabolite profiles to provide defensive functions against stresses or assist plant development and reproductive processes [1,2]. Phytohormones are key links between insect attack and the formation of the specialized metabolites. Jasmonic acid (JA) is mostly reported as being involved in interactions between insects and plant vegetative parts [3], while ethylene is mainly known to be responsible for interactions between insects and plant floral parts [4]. Herbivore attacks on plant vegetative parts generally increase JA synthesis, leading to upregulated expression of genes involved in the biosynthesis of specialized metabolites with antiherbivore function [5]. Pollination of plant floral parts could lead to an increase in ethylene synthesis that downregulates expression of volatile-related genes and thus reduces the emission of floral volatiles [4]. Such quantitative postpollination changes in floral bouquets help direct pollinators to unpollinated flowers and thus enhances the overall reproductive success [6]. Besides ethylene, it remains to be determined if other phytohormones such as JA play an important role in interactions between insects and plant floral parts. Additionally, most studies have focused on model plants so less attention has been paid to horticultural crops.

Tea (*Camellia sinensis*) plants are famous horticultural and representative plants in China. The leaves are the most intensively studied part because they have long been used to make tea. In recent years, the flowers of tea plants have attracted increasing interest because they contain functional molecules similar to those found in tea leaves but at higher levels [7]. Some representative metabolites in tea leaves, including catechins, flavonols, caffeine, and amino acids (for example, theanine) are also found in tea flowers [8,9,10]. Tea flowers also contain greater amounts of some metabolites that occur in tea leaves in lower or trace amounts such as floratheasaponins, spermidine derivatives, acetophenone, and 1-phenylethanol [11,12,13,14,15,16,17]. As a potentially rich resource, the chemical profiles and bioactivities of tea flowers have been intensively studied [7]. Tea flowers are generally available from September to December (autumn and winter seasons). Tea leaves are available during the whole year, but tender tea leaves are harvested for making tea products in spring, summer, and autumn seasons, respectively. During the growth of tea leaves, many pest insects attack tea leaves and affect the yield and quality of tea leaves. In response to insect attacks, tea plants emit numerous volatile compounds [18]. Some insect-induced volatiles may play important roles in defense [19,20]. Similar to other plants, formation of these insect-induced volatiles in tea leaves were related to JA induced by insect attacks [18,21]. Understanding formation of defensive metabolites in tea leaves exposed to insect attacks will contribute to the future development of the biological control of pest insects in tea plants. Plant defense against insects is generally a systematic defense. Therefore, besides local defense such as investigations from tea leaves, systematic defense, for example investigations from other tissues such as tea flowers, is required, although it remains to be determined if insects attacking tea flowers also affect quality and yield of tea leaves. In our previous study, we found that insect attack (*Thrips hawaiiensis* (Morgan)) can change the stereochemical configuration of 1-phenylethanol emitted from tea flowers in a manner that was not related to ethylene but may be associated with JA [16]. This allows us to use tea flowers as a model for studying the involvement of JA in interactions between insects and flowers. In the present study, *allene oxide synthase 2* (*AOS2*), which was found to be the only JA synthesis gene upregulated in tea flowers exposed to *Thrips hawaiiensis* (Morgan) attacks [16], was functionally characterized. Furthermore, the effect of JA on metabolite profiles and ethylene formation in tea flowers were also investigated. The information will advance our understanding of the involvement of phytohormones in interactions between flowers of horticultural crops and insects.

## 2. Results

### 2.1. Insect Attack-Upregulated CsAOS2 Functions in JA Synthesis in Tea Flowers

#### 2.1.1. Phylogenetic Analysis of CsAOSs and Their Subcellular Localization

**Figure 1:**
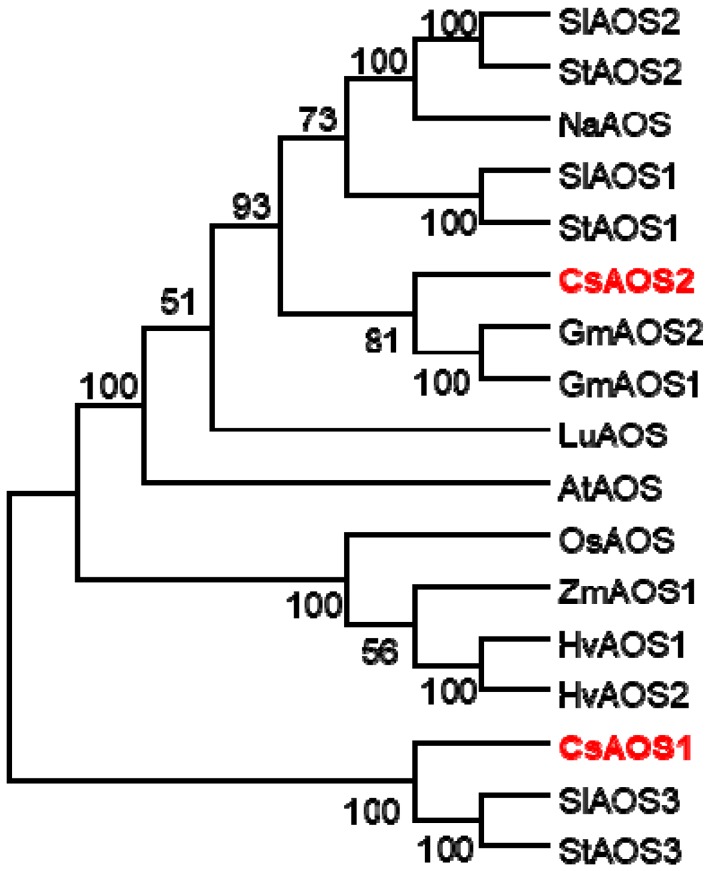
Phylogenetic analyses of plant allene oxidase synthases (AOSs) related to the function of jasmonic acid (JA) synthesis. Phylogenetic tree was performed by software MEGA 5.1 with neighbour joining computation. Sl: *Solanum lycopersicum*; St: *Solanum tuberosum*; Na: *Nicotiana attenuata*; Cs: *Camellia sinensis*; Gm: *glycine max*; Lu: *Linum usitatissimum*; At: *Arabidopsis thaliana*; Os: *Oryza sativa*; Zm: *zea mays*; Hv: *Hordeum vulgare*. The protein sequences of AOS were SlAOS2 (NP_001274707.1), StAOS2 (CAD29736.2), NaAOS (CAC82911.1), SlAOS1 (CAB88032.1), StAOS1 (CAD29735.1), CsAOS2 (AHY03308.1), GmAOS2 (NP_001236445.1), GmAOS1 (NP_001236432.1), LuAOS (P48417.1), AtAOS (CAA63266.1), OsAOS (AAL17675.1), ZmAOS1 (NP_001105244.2), HvAOS1 (CAB86383.1), HvAOS2 (CAB86384.1), CsAOS1 (BAU24784.1), SlAOS3 (NP_001265949.1), and StAOS3 (CAI30876.1).

In our previous study, we investigated the effects of *T. hawaiiensis* treatments on the expression levels of JA synthesis-related genes in tea flowers. Only *CsAOS2* expression was upregulated by *T. hawaiiensis* treatment, while other JA synthesis genes were not significantly affected [16]. To further validate the function of CsAOS2 in JA synthesis in tea flowers, in the present study, we first compared the homologous protein sequences of AOSs among different plant species, including GmAOS1 (*Glycine max*), GmAOS2, HvAOS1 (*Hordeum vulgare*), HvAOS2, SlAOS1 (*Solanum lycopersicum*), SlAOS2, SlAOS3, StAOS1 (*Solanum tuberosum*), StAOS2, StAOS3, NaAOS (*Nicotiana attenuata*), CsAOS1 (*C. sinensis*), CsAOS2, LuAOS (*Linum usitatissimum*), AtAOS (*Arabidopsis thaliana*), OsAOS (*Oryza sativa*), and ZmAOS1 (*Zea mays*) [22,23,24,25] (Figure 1). The phylogenetic analysis indicated that CsAOS1 had lower similarity with CsAOS2, which only showed 55.01% (Figure 1).

**Figure 2:**
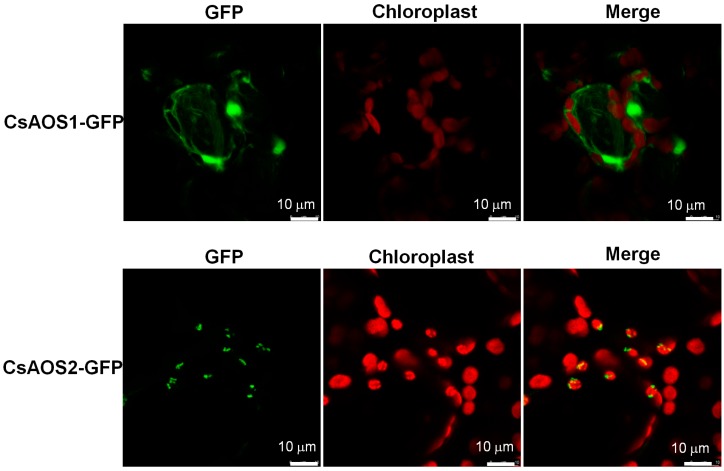
Subcellular localizations of CsAOS1 and CsAOS2. The CsAOS1-GFP and CsAOS2-GFP fusion proteins were transiently expressed in *Nicotiana benthamiana*. Green fluorescence indicated CsAOS1-GFP and CsAOS2-GFP fusion protein. Red fluorescence showed the chloroplast auto-fluorescence.

It was reported that wounding causes the release of linoleic acid (LA) from chloroplast lipids, catalyzed by lipoxygenases (LOX), AOS, and allene oxide cyclase (AOC) to 12-oxo-phytodienoic acid. 12-oxo-Phytodienoic acid produced in the chloroplast is transported to the peroxisome where it is used to synthesize JA [26,27,28,29]. In the present study, we investigated the subcellular localization of CsAOS1 and CsAOS2 by GFP-fusion protein pCAMBIA3300. CsAOS1 was located in the plasma membrane and CsAOS2 was detected in the chloroplast membrane (Figure 2). 

#### 2.1.2. Transient Expression Analysis in *N. benthamiana* Plants Confirmed That CsAOS2 Functioned in JA Synthesis

**Figure 3:**
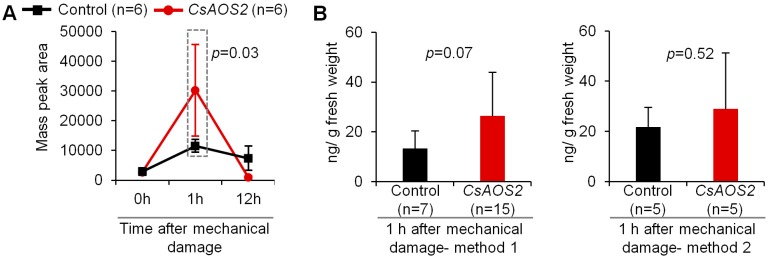
Comparison of JA contents in control and overexpression of CsAOS2 in *N. benthamiana*. (**A**) The treated leaves were harvested at the indicate time points 0 h, 1 h, and 12 h (*n* = 6), after mechanical damage; (**B**) Mechanical damage method 1 was damage by making 12 holes on one leaf using needle. Mechanical damage method 2 was damage by cutting one leaf into eight pieces. The treated leaves were harvested at 1 h, after mechanical damage. Data represent the mean value ± standard deviation.

To further confirm whether CsAOS2 functions in JA synthesis, transient expression analysis in *N. benthamiana* plants was employed (Figure 3). As the JA synthetic pathway derived from LA involves several enzymes/genes, the overexpression of individual genes may be insufficient to increase JA content. Therefore, mechanical damage was used to activate the genes in the JA synthetic pathway. In the present study, there were two groups of *N. benthamiana* plants including control (vector) and *CsAOS2* overexpression plants. These two groups of plants were treated by mechanical damage. As shown in Figure 3A, JA content in the control increased at 1 h after mechanical damage (damage by making 12 holes on one leaf using needle) because of upregulation of the genes in the JA synthetic pathway. Furthermore, the *CsAOS2* overexpression group had higher JA content than the control group at 1 h after the mechanical damage, indicating that CsAOS2 functions in JA synthesis. At 12 h after mechanical damage, JA content in both groups decreased; this was reasonable given that the JA response is relatively quick when plants are exposed to stresses [30,31]. We also investigated the effect of mechanical damage treatments of different intensities on functional characterization of *CsAOS2* at 1 h after mechanical damage. Figure 3B shows the two mechanical damage methods. The first method involved prodding 12 holes in each leaf using a needle. The second method involved cutting the leaves into eight parts. Different treatments led to different levels of JA increase in the *CsAOS2* overexpression group. This is because the intensity of the mechanical damage can affect the degree of expression changes observed for several genes in the JA synthetic pathway; these variation ranges may be higher than the overexpression level of *CsAOS2*. 

### 2.2. In Contrast to Tea Leaves, the Metabolite Profiles of Tea Flowers Were Not Significantly Affected by JA Treatment

**Figure 4:**
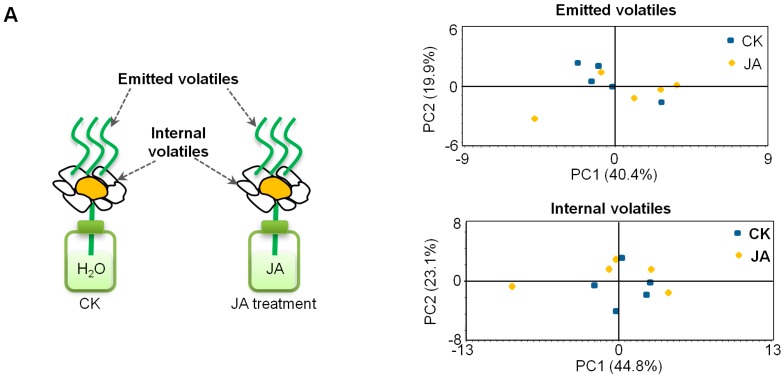

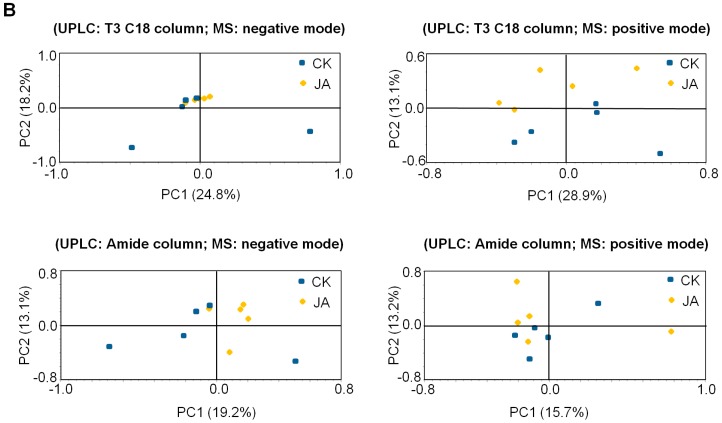
Effect of JA treatment on metabolite profiles of tea flowers. (**A**) JA treatment: 2.5 mM JA dissolved in 0.5% ethanol; control: distilled water dissolved in 0.5% ethanol. Principal component analysis (PCA) of emitted volatiles and internal volatiles, which were analyzed by GC-MS (*n* = 5). (**B**) PCA analysis of nonvolatile metabolites, which were analyzed by UPLC-QTOF-MS (*n* = 5).

**Figure 5:**
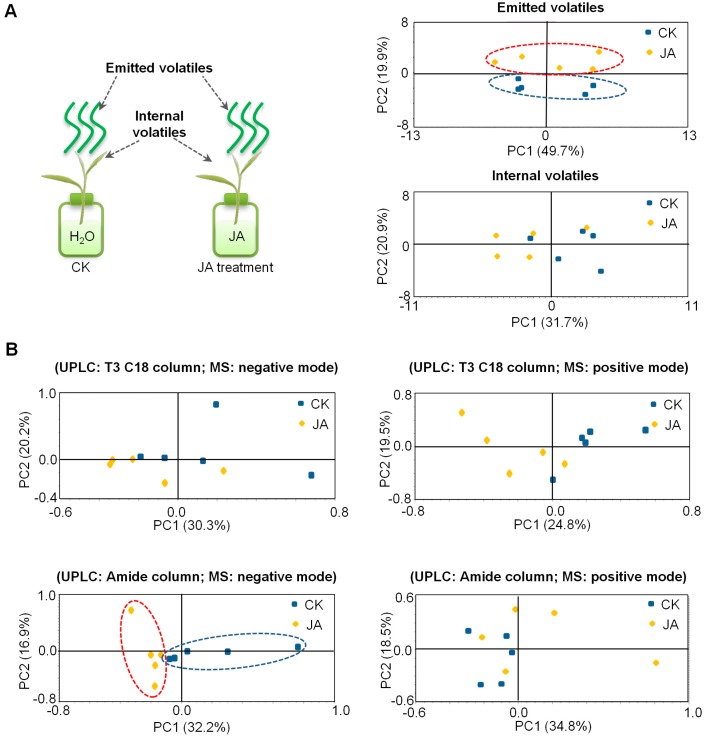
Effect of JA treatment on metabolite profiles of tea leaves. (**A**) JA treatment: 2.5 mM JA dissolved in 0.5% ethanol; control: distilled water dissolved in 0.5% ethanol. Principal component analysis (PCA) of emitted volatiles and internal volatiles, which were analyzed by GC-MS (*n* = 5). (**B**) PCA analysis of nonvolatile metabolites, which were analyzed by UPLC-QTOF-MS (*n* = 5).

It is well-known that JA can affect metabolite profiles in plants, especially vegetative parts, for example, the secondary metabolites in *Nicotiana attenuata* leaves [32]. To investigate whether JA affect the metabolite profiles in tea flowers, tea flowers were treated with JA (2.5 mM) for 10 h (from flower half-open stage to fully-open stage) and metabolite profiles were subsequently analyzed by gas chromatography-mass spectrometry (GC-MS, for volatile metabolites) and ultra-performance liquid chromatography/quadrupole time-of-flight mass spectrometry (UPLC-QTOF-MS, for nonvolatile metabolites). Emitted volatile and internal volatile profiles were not significantly changed in JA-treated tea flowers (Figure 4A). In the present study, UPLC–QTOF–MS was equipped with either an ACQUITY UPLC BEH Amide column (suitable for determination of high polarity metabolites) or an ACQUITY UPLC HSS T3 C18 column (suitable for determination of low polarity metabolites). In analyses with separations using both columns and positive or negative MS modes, nonvolatile metabolite profiles were also not significantly changed in JA-treated tea flowers (Figure 4B). In contrast, we also investigated the effects of the same JA treatment on metabolite profiles in tea leaves. Unlike tea flowers, volatile metabolite profiles and partial nonvolatile metabolite profiles of tea leaves were changed (Figure 5). These results suggest that the responses of metabolites to JA in tea flowers was weaker than in tea leaves. 

### 2.3. JA Treatment Did Not Influence Ethylene Formation in Tea Flowers

**Figure 6:**
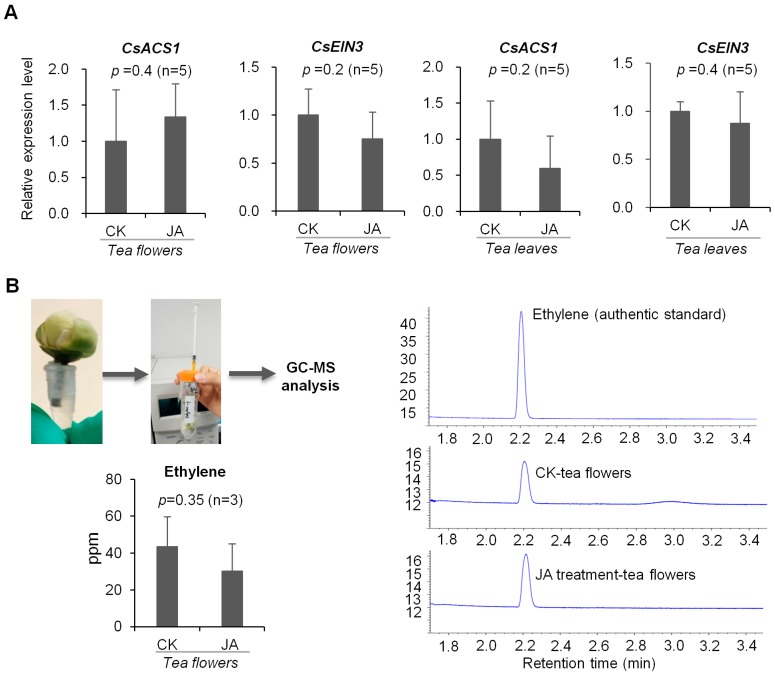
Effect of JA treatment on ethylene expression level and emission in tea flowers. CK, tea flowers without JA treatment. (**A**) Expression levels of ethylene synthesis genes and transcription factor in tea flowers and leaves, respectively which were treated by JA. Tea flowers and leaves were *C. sinensis* cv. Jinxuan. (**B**) The content of flowers ethylene were collected and analysis was conducted by GC. Tea flowers were *C. sinensis* cv. Yinghong NO. 9.

It has been reported that JA can enhance ethylene formation in *dendrobium* and *petunia* flowers [33]. Therefore, we also investigated whether JA in tea flowers has a similar effect on ethylene formation. *S*-Adenosyl-l-methionine synthetase (SAM) converts methionine into *S*-adenosyl-l-methionine (*S*-AdoMet), the precursor of ethylene. This is followed by ethylene biosynthesis. In the first step, *S*-AdoMet is converted into 1-aminocyclopropane-1-carboxylic acid (ACC) by ACC synthase (ACS). ACC oxidase (ACO) then oxidizes ACC to ethylene [34]. The key genes involved in ethylene formation, including a structural gene *ACS* and an ethylene receptor *ethylene-insensitive* (*EIN*) gene, found in *C. sinensis* were analyzed. In flowers of *C. sinensis* cv. Jinxuan (middle and small leaf species), the expression levels of these ethylene synthesis-related genes were not significantly affected by JA treatment (Figure 6A). To further confirm the relationship between ethylene and JA we measured the ethylene levels released from flowers (a big leaf species cv. Yinghong NO. 9) after JA treatment to exclude the effects of different tea cultivars (Figure 6B). Like ethylene synthesis-related gene expression levels, ethylene content was not significantly affected by JA treatment (Figure 6B). These results suggest that JA in tea flowers may have different functions than in the flowers of *dendrobium* and *petunia* [33].

## 3. Discussion

JA is widely present in the plant kingdom and belongs to the family of oxygenated fatty acid derivatives. It is synthesized via the oxidative metabolism of polyunsaturated fatty acids [35]. LA is oxygenated to 13(*S*)-hydroxy linolenic acid (13-HPOT) by the action of LOX. Under the actions of AOS and AOC, 13-HPOT then can be converted to 12-oxophytodienoic acid that can then be transformed to JA through reduction and three steps of β-oxidation. Among the enzymes in the JA synthesis pathway, LOXs are the most intensively studied enzymes. Plant LOXs are divided into two types including 9-LOXs and 13-LOXs. In the model plant *N. attenuata*, three different LOX isoforms are known. NaLOX3 is confirmed to be involved in the JA pathway and NaLOX2 has been shown to the pathway for green leaf volatiles [36,37]. In response to herbivore attack, upregulation of *LOX3* in *N. attenuata* leaf is more sensitive than other genes involved in the JA pathway [5]. There are many reports concerning the effect of herbivore attack on genes in the JA synthesis pathway in plant vegetative parts, while little corresponding information is available for plant floral parts. Tea plants have no successful genetic transformation system, making it difficult to obtain in vivo evidence of metabolic pathways in tea plants. Our current knowledge concerning phytohormone and metabolite biosynthesis in tea plants is mostly based on reported findings for other plant species. Although some of these pathways are shared among plant species, there may also be variation among species because of the complexity of the biosynthetic networks. Therefore, direct investigations of JA synthesis in tea plants are required. In the present study, CsAOS2 was confirmed to be involved in the biosynthesis of JA in tea flowers (Figure 3). The AOSs have been isolated and functionally characterized in several species of plants such as *Lycopersicon esculentum*, *Hordeum vulgare*, and *Solanum tuberosum*. These AOSs were reported to be located in the chloroplasts [26,27,28,38]. These results suggest that CsAOS2, rather than CsAOS1, may be involved in JA biosynthesis in tea flowers. In future work, it would be of interest to investigate why *CsAOS2* was upregulated in tea flowers exposed to *T. hawaiiensis* treatment while other genes were not significantly affected. 

There are a number of studies on the involvement of phytohormones in interactions between insects and flowers or leaves. Although some cases may be different in different plants, it is relatively universal that in most plants JA is typically reported to be involved in interactions between insects and plant vegetative parts while ethylene is mostly reported to be involved in interactions between insects and plant floral parts. Salicylic acid is mainly reported to be involved in interactions between microorganisms and plants, but it is also related to the interaction between insects and plant vegetative parts in some cases. More evidence suggests that, rather than individual phytohormones, interactions between insects and plants involve several phytohormones working together. Moreover, the phytohormone pathways may have antagonistic/synergistic cross-talk. For example, in maize, fungal infection reduced the emission of volatiles induced by lepidopteran herbivory alone by about 50%, possibly suggesting a diversion of plant resources from antiherbivore to presumptive antipathogen defenses. The authors hypothesized that fungal infection could stimulate the SA-based signal transduction pathway that would reduce signaling through the herbivore-triggered JA pathway because of negative cross-talk [39]. In contrast to plant vegetative parts, phytohormone cross-talk in plant floral parts is less studied. There are a few reports that JA can affect ethylene formation in flowers of dendrobium and petunia [33]. However, in the present study, JA did not significantly affect ethylene formation in tea flowers (Figure 6), suggesting that JA may play different roles in different plant flowers. Additionally, in contrast to tea leaves, the metabolite profiles of tea flowers were not significantly affected by JA treatment (Figure 4 and Figure 5). This also suggests that JA may have different effects in different tissues of the same plant. In tea leaves, JA treatment mainly increased emission of volatiles compounds such as α-farnesene, β-ocimene, (*Z*)-3-hexen-1-ol, linalool, benzyl alcohol, benzyl nitrile, indole, etc. [18,21]. Some volatiles emitted from tea leaves can attract natural enemies of the pest insects [19,20]. In our previous study, JA can change the stereochemical configuration of 1-phenylethanol emitted from tea flowers [16]. It will be of interest to investigate whether JA is also associated with the alteration of certain metabolites in tea flowers. Recently, Li et al. uncovered the defensive function of JA signaling in *N. attenuata* flowers that includes components that tailor JA signaling to provide flower-specific defenses [40]. Further studies on the relationship between insect-induced JA increase in tea flowers and their defense against insect attacks will help us further understand the roles of flowers in tea plants and systematic defense of tea plants. 

## 4. Materials and Methods

### 4.1. Plant Materials and Treatments

The flowers and leaves of *C. sinensis* cv. ‘Jinxuan’ and flowers of cv. ‘Yinghong No. 9’ were sampled from the Tea Research Institute, Guangdong Academy of Agricultural Sciences (23° N, 113° E, Yingde, China).

For phytohormone treatments, half-open flowers and a bud with two leaves were placed in solution A and solution B, respectively. Solution A contained 2.5 mM JA dissolved in 0.5% ethanol (*v*/*v*), solution B contained distilled water with 0.5% ethanol (*v*/*v*). All treatments were performed for 10 h at room temperature. Groups of three treated flowers and groups of five treated leaves were used as one replicate, respectively, for collecting volatile emissions. Groups of five flowers or leaves were collected as one replicate for internal volatiles, nonvolatile metabolites, and phytohormone analyses, respectively. Each treatment was performed in quintuplicate. 

### 4.2. Extraction and Analysis of Volatile Metabolites

For extraction of internal volatile metabolites, the treated flowers were ground with liquid nitrogen before 0.5 g of finely powdered flowers was extracted in 5 mL CH_2_Cl_2_; 50 μL 0.1 mM ethyl *n*-decanoate was used as an internal standard. Extractions were shaken for 4 h at room temperature. The process was the same as previously described [16]. The extraction was then dried over anhydrous sodium sulfate and concentrated to 200 μL using nitrogen gas before 1 μL of the concentrate was subjected to analysis by GC−MS.

For the collection of emitted volatile metabolites, three flowers, or five tea shoots (one bud and two leaves per shoot), were placed into 1-L beakers and then sealed with silver paper. The emitted volatiles were collected as previously described [16]. The headspace volatiles were absorbed by solid phase microextraction (SPME, 50/30 μm, DVB/CAR/PDMS, Stableflex (2 cm)) (Supelco Inc., Bellefonte, Pennsylvania, USA) for 10 min (for tea flowers) and 30 min (for tea leaves), respectively, at room temperature and then analyzed by GC−MS. 

The GC-MS was equipped with a SUPELCOWAX™ 10 column (Supelco Inc., 30 m × 0.25 mm × 0.25 μm). The injector temperature was 230 °C, the split ratio was 5:1 (for flower internal volatiles), split 10:1 (for flower-emitted volatiles), and splitless (for tea leaf-emitted and internal volatiles); the helium (carrier gas) flow rate was 1.0 mL/min. The initial temperature of the column was 60 °C, maintained for 3 min, followed by a ramped temperature rise to 240 °C at a rate of 4 °C/min before being held at 240 °C for 20 min. Full scan mode (mass range *m*/*z* 40−200) was operated for MS analysis.

### 4.3. Extraction and Analysis of Nonvolatile Metabolites

One hundred mg of finely powdered flowers was extracted with 3 mL cold 70% methanol using ultrasonic extraction for 30 min under ice-cold conditions. The extractions were centrifuged for 10 min at 10,000× *g* at 4 °C to obtain the supernatant and then filtered through a 0.22 μm membrane. Samples were analyzed by UPLC-QTOF-MS (Waters Corporation, Milford, Massachusetts, MA, USA). Each sample (5 μL) was injected into a Waters ACQUITY UPLC HSS T3 C18 column (2.1 mm × 100 mm, 1.8 μm). Solvent A was Milli-Q water with 0.1% (*v*/*v*) formic acid. Solvent B was acetonitrile with 0.1% (*v*/*v*) formic acid. The solvent gradient was as follows: solvent B was started at 10% then linearly increased to 30% within 25 min, then increased to 95% within 15 min and kept for 4 min before being dropped to 10% in 0.1 min and maintained for 4 min. The flow rate was 0.3 mL/min and the column temperature was 30 °C. The electrospray ionization operated on positive and negative modes, respectively. The MS conditions of the positive mode were as follows: capillary voltage was 3 kV, source temperature was 100 °C, desolvation temperature was 350 °C, cone gas flow was 50 L/h, and desolvation gas flow was 650 L/h. The MS conditions of the negative mode were as follow: capillary voltage was 1.5 kV, source temperature was 100 °C, desolvation temperature was 300 °C, cone gas flow was 50 L/h, and desolvation gas flow was 600 L/h.

We also used an ACQUITY UPLC BEH Amide column (2.1 mm × 100 mm, 1.7 μm) to identify some polar compounds. Solvent A was Milli-Q water with 0.1% (*v*/*v*) formic acid. Solvent B was acetonitrile with 0.1% (*v*/*v*) formic acid. The solvent gradient was as follows: solvent B was started at 90%, then linearly decreased to 50% within 30 min and kept for 4 min before it was increased to 90% in 0.1 min and maintained for 4 min. The flow rate was 0.4 mL/min. The column temperature was 30 °C. The electrospray ionization operated on positive and negative modes, respectively. The MS conditions of the positive mode were as follows: capillary voltage was 2 kV, source temperature was 100 °C, desolvation temperature was 350 °C, cone gas flow was 50 L/h, and desolvation gas flow was 600 L/h. The MS conditions of the negative mode were as follows: capillary voltage was 0.5 kV, source temperature was 100 °C, desolvation temperature was 250 °C, cone gas flow was 50 L/h, and desolvation gas flow was 400 L/h.

### 4.4. Gene Cloning

Homologous genes of *AOS* were identified using *C. sinensis* transcriptome information. RNA was then isolated from 150 mg of flowers (cv. Jinxuan) using a Quick RNA Isolation Kit (Huayueyang, Beijing, China) and 20 μL of cDNA was synthesized from 500 ng of total RNA using a Prime Script^TM^ RT reagent Kit with gDNA Eraser (Takara Biotechnology, Dalian, China) according to the manufacturer’s instructions. Reverse transcription was carried out at 37 °C for 15 min, followed by 85 °C for 5 s. Genes were obtained by polymerase chain reaction (PCR) using this cDNA as the template. We use nested PCR to successfully obtain the sequences. Two rounds of PCR primers are listed in Tables S1 and S2. The conditions of the first reaction were as follows; 98 °C for 2 min followed by 35 cycles of 98 °C for 10 s, 57 °C for 15 s, and 72 °C for 1 min before samples were maintained at 16 °C. The second reaction consisted of 98 °C for 2 min followed by two cycles of 98 °C for 10 s, 47 °C for 15 s, and 72 °C for 1 min. This was followed by 32 cycles of 98 °C for 10 s, 61 °C for 15 s, and 72 °C for 1 min before the samples were maintained at 16 °C. Products were gel purified, cloned into the pGEM-T easy vector with T4 ligase, and sequenced. The phylogenetic analyses were performed by MEGA 5.1 software using the neighbor-joining method.

### 4.5. Subcellular Location Analysis of CsAOSs

The complete sequence of the *CsAOS* ORF was cloned and inserted into pCambia3300-GFP vectors using an In-Fusion^®^ HD Cloning Kit (Takara Biotechnology. The construct was transformed into *Agrobacterium tumefaciens* strain GV3101 by chemical transfection. One single colony of *Agrobacterium* was then inoculated into 5 mL LB with appropriate antibiotics (Rif, Gen, and Kana) and grown overnight at 28 °C. Subsequently, 1 mL of the overnight culture was added to 25 mL LB plus 20 μM acetosyringone and grown again overnight. The bacteria were precipitated at 5000× *g* for 8 min before the pellet was resuspended in resuspension buffer (10 mM MgCl_2_, 10 mM MES-K (pH 5.6), and 100 μM acetosyringone). The final A600 was be adjusted to 0.4 nm and samples were left at room temperature for at least 2 h before being infiltrated into *N. benthamiana* leaves using a needle-less syringe. After 5 days, the fluorescence-labeled protein was observed under a confocal laser-scanning microscope Zeiss LSM 510 (Carl Zeiss, Jena, Germany).

### 4.6. Analysis of the Activities of CsAOS2 in N. benthamiana Overexpression Lines

*CsAOS2* was transiently expressed in *N. benthamiana* as described above. Leaves from these plants were mechanically damaged using a needle before the treated leaves were harvested at the indicated time points (0 h, 1 h, and 12 h) and immediately frozen in liquid nitrogen. Next, two types of mechanical damage were applied to analyze the function of CsAOS2 1 h after treatments. One method involved needle puncture while in the other the leaf was cut into eight pieces. In each instance, samples were then immediately frozen in liquid nitrogen. 

Next, 300 mg of finely powdered leaves was extracted with 2 mL cold ethyl acetate. The extraction was vortexed for 30 s and then ultrasonically extracted for 20 min under ice-cold conditions. Subsequently, the solution was centrifuged at 12,000× *g* at 4 °C for 10 min to obtain the supernatant. Next, 1 mL ethyl acetate was added to the residue and the samples were vortexed for 30 s before being ultrasonically extracted for 10 min under ice-cold conditions. The supernatants were combined and dried under nitrogen gas. Dried residues were resuspended in 200 μL methanol containing 40 ng of [^2^H_5_]-JA (2, 4, 4-d3; acetyl-2, 2-d2) as an internal standard and then passed through a 0.22 μm filter membrane.

The extraction was analyzed by UPLC-QTOF-MS (Waters Corporation, Milford, USA) running in the MS^2^ acquisition mode. Each sample (5 μL) was injected onto a Waters ACQUITY UPLC HSS T3 C18 column (2.1 mm × 100 mm, 1.8 μm). Solvent A was Milli-Q water with 0.1% (*v*/*v*) formic acid and solvent B was acetonitrile with 0.1% (*v*/*v*) formic acid. The solvent gradient was as follows: solvent B was started at 20% then linearly increased to 35% within 10 min, and then increased to 95% in 0.1 min and maintained for 3 min before being dropped to 20% in 0.1 min and maintained for 3 min. The flow rate was 0.4 mL/min and the column temperature was 30 °C. The electrospray ionization operated on negative mode. The MS conditions were as follows: capillary voltage was 1.5 kV, source temperature was 100 °C, desolvation temperature was 300 °C, cone gas flow was 50 L/h, and desolvation gas flow was 600 L/h.

### 4.7. Transcript Expression Analyses of Genes in Ethylene Formation in Tea Flowers

RNA was isolated from 150 mg of flowers (cv. Jinxuan) and 100 mg of leaves (cv. Jinxuan) using a Quick RNA Isolation Kit (Huayueyang, Beijing, China). Then, 20 μL of cDNA was synthesized from 500 ng of total RNA using Prime Script^TM^ RT reagent Kit with gDNA Eraser (Takara Biotechnology, Dalian, China) according to the manufacturer’s instructions. Reverse transcription was carried out at 37 °C for 15 min, followed by 85 °C for 5 s. The primers used in the quantitative real-time PCR (qRT-PCR) are listed in Table S3. Elongation factor-1a (*EF-1a*) was used as an internal reference. 

The qRT-PCR analysis reactions were performed in a total volume of 20 μL, including 0.8 μL of each primer (10 μM), 2 μL cDNA diluted 50-fold, 10 μL iTaq^TM^ Universal SYBR^®^ Green Supermix (Bio-Rad, California, USA), and 6.8 μL ddH_2_O. The reactions were performed using a Roche LightCycler 480 (Roche Applied Science, Mannheim, Germany). The PCR program was initiated with a preliminary step of 5 min at 95 °C, followed by 45 cycles of 95 °C for 15 s and 60 °C for 1 min. A melting curve was generated for each sample at the end of each run to ensure the purity of the amplified products. The 2^−ΔΔ*C*t^ method was used to calculate fold change as previously described [16].

### 4.8. Collection and Analysis of Ethylene in Tea Flowers

For the collection of emitted ethylene, each flower was placed in a 200 μL centrifuge tube with 2.5 mM JA dissolved in 0.5% ethanol (*v*/*v*); nine of these flowers were then placed in a 50 mL centrifuge tube that was then sealed with silver paper. Air in the tube was extracted using a 1-mL syringe. These 1-mL gas samples were analyzed with a GC equipped with a HP-PLOT/Q column (30 m × 0.32 mm × 20 μm, Agilent Technologies, Wilmington, DE, USA).

The GC front detector FID was heated to 250 °C, and the fuel gas (hydrogen) flow was set to 30 mL/min to maintain the flame. The air flow was set to optimum at 400 mL/min and the makeup flow was set at 25 mL/min. The column was used at an initial temperature of 60 °C maintained for 2 min. After the temperature reached equilibrium for 0.5 min, the oven was heated at a rate of 5 °C/min until it reached 150 °C; the temperature was then maintained for 1 min. The oven max temperature could not exceed 250 °C and the total GC run time was approximately 5 min. All GC measurement results were prepared using the Agilent Integrated GC Software.

### 4.9. Statistical Analysis

Excel Ver. 2013 software was used for statistical analysis. Student’s *t*-tests were used to identify significant differences between two treatment groups. A probability level of 5% (*p* ≤ 0.05) indicates a significant difference.

## 5. Conclusions

**Figure 7:**
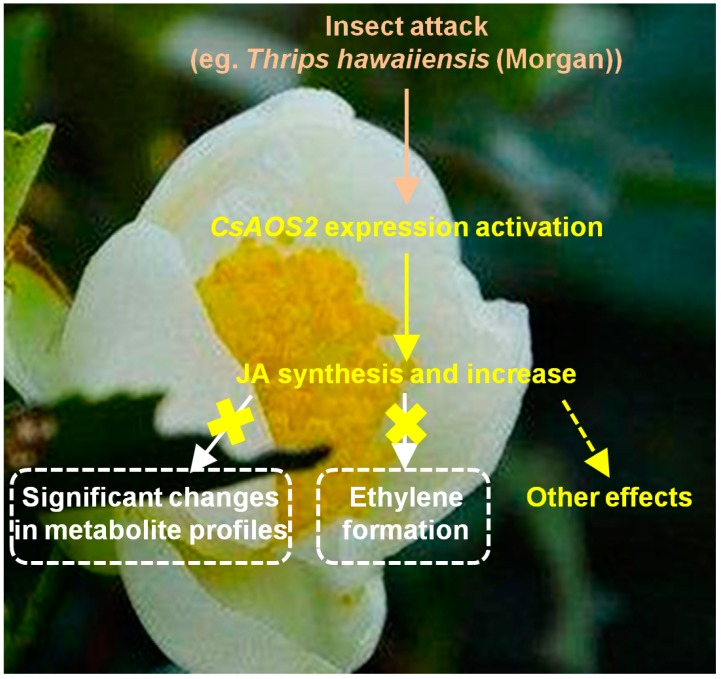
Summary on effect of insect (*Thrips hawaiiensis* (Morgan)) attack on pathway of JA and its influence on tea flowers.

As *CsAOS2* was found to be a specifically upregulated JA synthesis gene in tea flowers exposed to *T. hawaiiensis* attack [16], we provided further evidence that CsAOS2 functions in JA synthesis using *N. benthamiana* transient expression analysis and subcellular localization analysis. Moreover, in this study, JA in tea flowers showed different functions from tea leaves and other flowers and did not significantly influence the metabolite profiles of tea flowers and the formation of ethylene; this suggests that JA might instead have other functions such as changing stereochemical configurations (Figure 7). The information presented here will help us determine the involvement of phytohormones in interactions between the flowers of horticultural crops such as tea plants and insects.

## Supplementary Materials

Supplementary materials can be found at http://www.mdpi.com/1422-0067/19/8/2440/s1.

## Abbreviations

AOS	Allene oxide synthase
JA	Jasmonic acid
LA	Linoleic acid
LOX	Lipoxygenases
AOC	Allene oxide cyclase
AdoMet	*S*-adenosyl-l-methionine
SAM	*S*-adenosyl-l-methionine synthetase
ACC	1-Aminocyclopropane-1-carboxylic acid
ACS	ACC synthase
ACO	ACC oxidase
EIN	Ethylene-insensitive
13-HPOT	13(*S*)-hydroxy linolenic acid
GC-MS	Gas chromatography-mass spectrometry
UPLC-QTOF-MS	Ultra-performance liquid chromatography/quadrupole time-of-flight mass spectrometry
