# Supplementary material for: Functional Characterization of An Allene Oxide Synthase Involved in Biosynthesis of Jasmonic Acid and Its Influence on Metabolite Profiles and Ethylene Formation in Tea (Camellia sinensis) Flowers
Source: Int J Mol Sci. 2018 Aug 18;19(8):2440. doi: 10.3390/ijms19082440 (PMC6121675; doi:10.3390/ijms19082440)
Supplement: Supplementary file 1 [file ijms-19-02440-s001.pdf]

SUPPLEMENTARY INFORMATION

Functional Characterization of *An Allene Oxide Synthase* Involved in Biosynthesis of Jasmonic Acid and Its Influence on Metabolite Profiles and Ethylene Formation in Tea (*Camellia sinensis*) Flowers

Qiyuan Peng [1, 2, †], Ying Zhou [1, †], Yinyin Liao [1,2], Lanting Zeng [1,2], Xinlan Xu [1], Yongxia Jia [1], Fang Dong [3], Jianlong Li [4], Jinchi Tang [4], Ziyin Yang [1,2,*]

[1] Guangdong Provincial Key Laboratory of Applied Botany & Key Laboratory of South China Agricultural Plant Molecular Analysis and Genetic Improvement, South China Botanical Garden, Chinese Academy of Sciences, Xingke Road 723, Tianhe District, Guangzhou 510650, China; pqyuan@scbg.ac.cn (Q.P.); yzhou@scbg.ac.cn (Y.Z.); honey_yyliao@scbg.ac.cn (Y.L.); zenglanting@scbg.ac.cn (L.Z.); xxl@scbg.ac.cn (X.X.); jyx@scbg.ac.cn (Y.J.)

[2] University of Chinese Academy of Sciences, No. 19A Yuquan Road, Beijing 100049, China

[3] Guangdong Food and Drug Vocational College, Longdongbei Road 321, Tianhe District, Guangzhou 510520, China; dongfangxyz@163.com (F.D.)

[4] Tea Research Institute, Guangdong Academy of Agricultural Sciences & Guangdong Provincial Key Laboratory of Tea Plant Resources Innovation and Utilization, Dafeng Road 6, Tianhe District, Guangzhou 510640, China; skylong.41@163.com (J.L.); tangjinchi@126.com (J.T.)

† These authors contributed equally to this work.

* Correspondence: zyyang@scbg.ac.cn; Tel./Fax: +86-20-3807-2989

Table S1. Primers of first round of AOS-PCR.

Gene	Forward primer 5'-3'	Reverse primer 3'-5'
AOS1	CCCCAAACCCAAGCAATGT	TGATTTACCACGGCAAGTG
AOS2	CCCCACAGCCCATTTATCT	TGCGTTGGACCGCC

AOS1, allene oxide synthase1; AOS2, allene oxide synthase 2.

Table S2. Primers of second round of AOS-PCR.

Gene	Forward primer 5'-3'	Reverse primer 3'-5'
AOS1	AAAGGATCCATGTCTTCTTCATCTATTTC	GGGACTAGTAGTCCACGTAGCCTTAG
AOS2	AAAGGATCCATGGCATCATCTTCTTT	GGGCAAACTAGTAAAACTAGCTCTCTTCAGC

AOS1, allene oxide synthase 1; AOS2, allene oxide synthase 2.

Table S3. Primers of qRT-PCR used in this study.

Gene	Forward primer 5'-3'	Reverse primer 3'-5'
CsEF1	TTGGACAAGCTCAAGGCTGAACG	ATGGCCAGGAGCATCAAT GACAGT
CsACS1	TGAGAGGCGATAGAGTGACATT	GCTGCTTCCAAGGCTGATT
CsEIN3	GGTAAGGAAGGAGTTGATGCT	CCGCTTGGTATTTCGCTATTG

EF1, encoding elongation factor 1; ACS1, 1-aminocyclopropane-1-carboxylic acid synthase 1; EIN3, ethylene-insensitive 3;.
